# Copper-Nanoparticle-Induced Neurotoxic Effect and Oxidative Stress in the Early Developmental Stage of Zebrafish (*Danio rerio*)

**DOI:** 10.3390/molecules29112414

**Published:** 2024-05-21

**Authors:** Na Liu, Luyao Tong, Kunjie Li, Qiuxia Dong, Jieying Jing

**Affiliations:** 1 State Key Laboratory of Clean and Efficient Coal Utilization, Taiyuan University of Technology, Taiyuan 030024, China; naliu@sxu.edu.cn; 2 School of Life Science, Shanxi University, Taiyuan 030006, China; 3 Shanxi Huaxin Tonghui Clean Energy Co., Ltd., Taiyuan 030032, China; 4 Shanxi Huaxin Gas Energy Institute Co., Ltd., Taiyuan 030032, China

**Keywords:** copper nanoparticles, neurotoxicity, oxidative stress, inflammatory response, zebrafish

## Abstract

Copper nanoparticles (CuNPs) are extensively used in electronics, cosmetics, fungicides, and various other fields due to their distinctive qualities. However, this widespread usage can contribute to environmental contamination and heightened health risks for living organisms. Despite their prevalent use, the ecological impacts and biosafety of CuNPs remain inadequately understood. The present study aims to delve into the potential toxic effects of CuNPs on zebrafish (*Danio rerio*) embryos, focusing on multiple indexes such as embryonic development, neurotoxicity, oxidative stress, and inflammatory response. The results revealed a notable increase in the death rate and deformity rate, alongside varying degrees of decrease in hatching rate and heart rate following CuNPs exposure. Particularly, the frequency of spontaneous tail coiling significantly declined under exposure to CuNPs at concentrations of 500 µg/L. Furthermore, CuNPs exposure induced alterations in the transcriptional expression of GABA signaling pathway-related genes (*gabra1*, *gad*, *abat,* and *gat1*), indicating potential impacts on GABA synthesis, release, catabolism, recovery, and receptor binding. Additionally, CuNPs triggered oxidative stress, evidenced by disruption in superoxide dismutase (SOD), catalase (CAT), and glutathione peroxidase (GPx) activities, along with elevated malondialdehyde (MDA) levels. This oxidative stress subsequently led to a proinflammatory cascade, as demonstrated by the increased transcriptional expression of inflammatory markers *(il-1β*, *tnf-α*, *il-6,* and *il-8*). Comparative analysis with copper ion (provided as CuCl_2_) exposure highlighted more significant changes in most indexes with CuCl_2_, indicating greater toxicity compared to CuNPs at equivalent concentrations. In conclusion, these findings provide valuable insights into the toxic effects of CuNPs on zebrafish embryo development and neurotransmitter conduction. Furthermore, they present technical methodologies for assessing environmental and health risks associated with CuNPs, contributing to a better understanding of their biosafety and ecological impact.

## 1. Introduction

With the rapid development and extensive use of nanoparticles (NPs) in sectors like the food industry, diagnostic medicine, drug delivery, electronics, cosmetics, and chemical industry [1], concerns about the potential environmental hazards and health risks of NPs are dramatically increasing. Copper nanoparticles (CuNPs), an important type of manufactured NPs, have been broadly used in lubricants, polymers/plastics, metallic coating, electronics, printers, cosmetics, and fungicides [2,3,4,5], owing to their unique optical, antifungal, and electrical characteristics [6,7]. However, the widespread usage of CuNPs raises environmental release and human exposure risks, necessitating increased focus on their ecological and biosafety implications.

In vitro studies have demonstrated that CuNPs exhibited toxic effects on ovarian granulosa cells including reduced cell viability, decreased mitochondrial membrane potential, induced apoptosis rate, and induction of oxidative stress [8]. Similarly, copper oxide (CuO) NPs exhibit significant cytotoxicity on human placental cells [9] and bone marrow mesenchymal stem cells [10], with a potential for selective apoptosis induction in cancer cells [11]. In vivo studies reveal decreased body weight and uterus weight, along with regulated expression of caspase 3 and apelin receptor (APJ), in mice exposed to CuNPs [12]. Yang et al. also found that CuNPs could induce ovarian injury, apoptosis, follicular atresia, and mRNA expression alterations in rats [1]. Furthermore, CuNPs have been involved in neurodegenerative disorder process, such as Parkinson’s and Alzheimer’s diseases [13]. 

Previous studies have highlighted that the effect of NPs can vary based on factors like particle size, surface activity, surface coating, chemical composition, and more. For example, smaller CuO NPs have heightened biological effects against breast cancer and nosocomial infection bacteria [14]. Jiang et al. discovered that silver nanoparticles (AgNPs) and gold nanoparticles, when coated with antibodies, can modulate membrane receptor internalization, where the size of the NPs significantly affects receptor binding, activation, and subsequent protein expression [15]. However, until now our understanding of CuNPs’ implications for ecosystems and human health remains limited as compared to that of other metal nanoparticles like AgNPs. Moreover, research on CuNPs with a small particle size is scarce.

The zebrafish (*Danio rerio*) is a valuable vertebrate model for toxicological research due to its genetic and developmental similarities to human [16]. Its increasing use in nanoparticle toxicity is notable [17]. Zebrafish embryos, being robust and external developing, offer easy observation of complex biological processes and manipulation during development. Moreover, their rapid development makes them widely applicable in lifespan toxicity research [18]. Therefore, zebrafish embryos were utilized in the present study to explore the potential toxic effect of CuNPs with a small particle size (5–10 nm) on early development. Normally, the toxicity of CuNPs is thought to be a combined result of the intracellular nanoparticles and the leaching of dissolved copper ions (Cu^2+^) [4]. Hence, Cu^2+^ (provided as CuCl_2_) treatment was included to compare the toxicity difference between Cu^2+^ and CuNPs exposure.

## 2. Results and Discussion

### 2.1. Bioaccumulation of Cu

**Figure 1:**
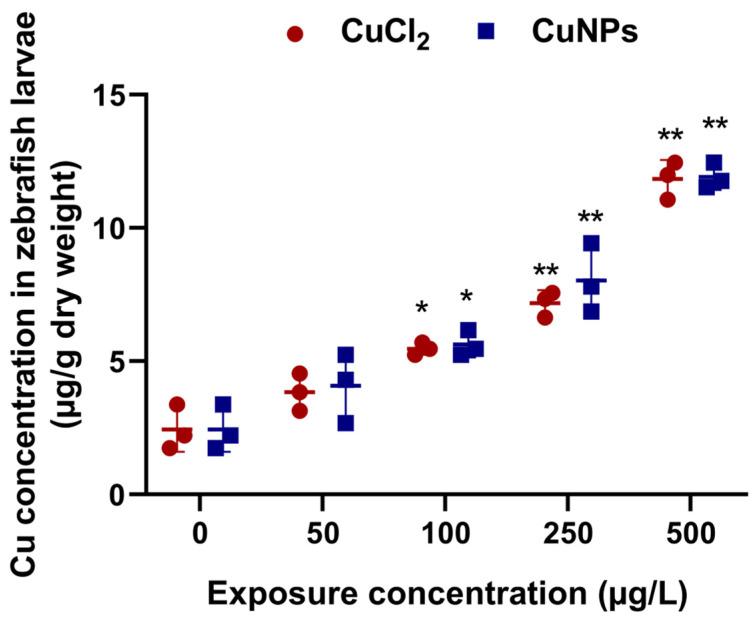
Cu concentration (µg/g dry weight) in the zebrafish embryos exposed to CuCl_2_ and CuNPs for 96 h. The experiment was performed in triplicate with N = 30 for each treatment. Data are mean ± SD. * *p* < 0.05, ** *p* < 0.01 versus the control.

After exposure for 96 h, the bioaccumulation of Cu in zebrafish larvae under different treatment is shown in Figure 1. Exposure to CuNPs and CuCl_2_ led to a substantial enhancement of Cu bioaccumulation in the zebrafish larvae, which increased significantly (*p* < 0.05) as the exposure concentration increased. Specifically, the bioaccumulation of Cu in zebrafish exposed to 500 µg/L CuNPs and CuCl_2_ increased by 4.87- and 4.83-fold, respectively, versus the control (without copper exposure). Interestingly, there was no significant difference in Cu concentration between the CuCl_2_ treatments and CuNPs treatments in zebrafish larvae.

Previous studies have also documented the bioaccumulation of certain NPs in aquatic animals. For example, rainbow trout (*Oncorhynchus mykiss*) that were exposed to serial concentrations (0.06, 0.6, and 6 µg/L) of AgNPs (particle size: 20 nm) and silver nitrate (AgNO_3_) for 96 h exhibited a significant increase in hepatic Ag content [19]. Zebrafish larvae exposed to AgNPs similarly showed a notable increase in Ag concentration after 21 days of exposure [20]. Likewise, exposure to CuNPs and CuSO_4_ caused a remarkable increase in Cu bioaccumulation in various tissues of red swamp crayfish (*Procambarus clarkii*) [21] and barnacle larvae (*Balanus amphitrite*) [22]. Compared to the control without Cu exposure, Cu bioaccumulation in barnacle larvae exposed to 200 µg/L CuNPs (particle size: 20 nm) increased by 5.3–12.6 times [22]. Wang et al. [23] also found an increased Cu level in juvenile, orange-spotted grouper (*Epinephelus coioides*) after exposure to both CuNPs and CuSO_4_. After ingestion, the NPs were distributed and accumulated in different tissues, which caused changes in physiological, behavioral, metabolic, and immunological functions [24,25,26,27].

### 2.2. Embryotoxicity Test

Zebrafish embryos undergo external and rapid development, maintaining robustness and transparency during this phase. These qualities make them easy to manipulate, suitable for high-throughput applications, and conducive to detailed visual analysis [28,29]. In the present study, a time- and concentration-dependent assessment was carried out during the embryonic stage of zebrafish to evaluate the developmental toxicity of CuNPs. Zebrafish embryos were exposed to CuNPs for 96 h, with daily recordings of death rate, hatching rate, heart rate, deformity rate, and frequency of spontaneous tail coiling.

**Figure 2:**
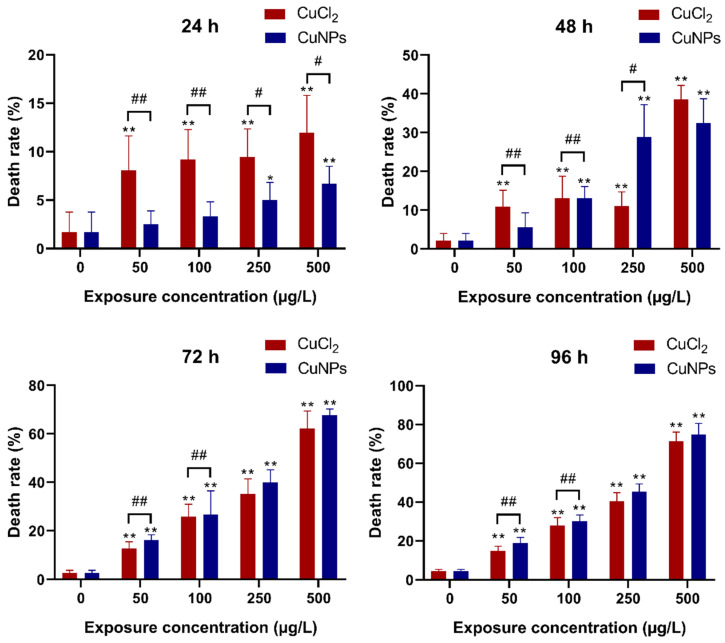
Death rate (%) of zebrafish embryos after treatment with different concentrations of CuCl_2_ and CuNPs for 96 h. Data are mean ± SD, N = 180. * Denotes significant difference versus the control (* *p* < 0.05, ** *p* < 0.01). # Denotes significant difference between CuCl_2_ and CuNPs groups (# *p* < 0.05, ## *p* < 0.01).

As shown in Figure 2, a remarkable enhancement of the death rate was observed in both CuNPs and CuCl_2_ treatments, correlating with the higher exposure concentration and longer duration. There were significant differences (*p* < 0.05) in the death rate between CuNPs and CuCl_2_ treatments after 24 h exposure, with increased death rate only seen at a high CuNPs concentration (250 and 500 µg/L). However, after 72 h and 96 h of exposure, the death rate significantly increased (*p* < 0.01) in both CuNPs and CuCl_2_ treatments, although no notable differences were noted between CuNPs and CuCl_2_ treatments at high concentration levels. In a toxicity study of iron oxide nanoparticles CR@Fe_3_O_4_, minimal toxic effects were found at high-dose treatment (800 μg/mL), leading to increased mortality and delayed hatching cycles [30]. Conversely, Lee et al. [31] found that exposure to polystyrene (PS) nanoplastics (0.1 mg/mL) had a negligible impact on zebrafish embryo mortality and deformation, indicating a lack of toxic effect from PS nanoplastics on the embryos.

**Figure 3:**
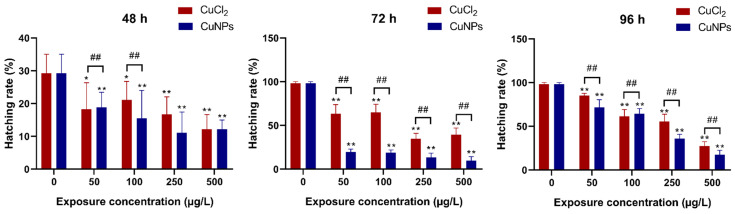
Hatching rate (%) of zebrafish embryos after treatment with different concentrations of CuCl_2_ and CuNPs for 96 h. Data are mean ± SD, N = 180. * Denotes significant difference versus the control (* *p* < 0.05, ** *p* < 0.01). ## Denotes significant difference between CuCl_2_ and CuNPs groups (*p* < 0.01).

Hatching is intricately regulated by various factors, encompassing both internal and external elements, and is highly sensitive to numerous environmental conditions and chemical substances. Therefore, changes in hatching patterns after nanomaterial exposure have been examined to gauge toxic effects on zebrafish previously [32,33,34,35,36,37,38]. In this study, the hatching rate increased at 72 and 96 h compared to 48 h for most concentrations of both CuCl_2_ and CuNPs. Conversely, a slight decrease in hatching rate was noted in embryos exposed to CuNPs and CuCl_2_ for 48 h (Figure 3). Significant impacts on the hatching rate (*p* < 0.01) were evident in embryos exposed for 72 and 96 h, with notable disparities observed between CuNPs and CuCl_2_ treatments across all the concentrations (*p* < 0.01). Previous research reported a decline in the hatching rate in zebrafish embryos exposed to Ag^+^ for 5 days, alongside significantly reduced embryonic survival and delayed hatching [32]. Similar findings were observed in a toxicity study involving cobalt ferrite (CoFe_2_O_4_) nanoparticles, revealing a dose- and time-dependent developmental toxic effect characterized by severe cardiac edema, hatching delay, tail/spinal cord flexure, and reduced metabolism [34].

**Figure 4:**
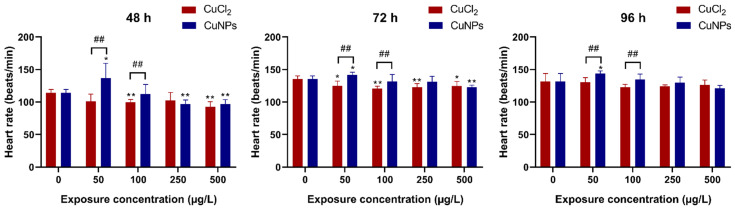
Heart rate (%) of zebrafish embryos after treatment with different concentrations of CuCl_2_ and CuNPs for 96 h. Data are mean ± SD, N = 60. * Denotes significant difference versus the control (* *p* < 0.05, ** *p* < 0.01). ## Denotes significant difference between CuCl_2_ and CuNPs groups (*p* < 0.01).

The heart of a zebrafish is a structurally similar model to the human heart and is easily accessible for optical analyses owing to the transparency in the larval stage, making it a prevalent choice for cardiac research. The heart rate serves an important parameter in both embryonic development and cardiac function assessment, as deviations in rhythm can be indicative of a developmental disorder or underlying cardiac pathologies [39]. In the present study, the heartbeats were calculated for samples treated with varying concentrations of CuNPs or CuCl_2_ at 48, 72, and 96 h (Figure 4). At 48 and 72 h, both CuNPs and CuCl_2_ treatments resulted in a decreased heart rate, except for a slight increase observed at 50 µg/L CuNPs, which was thought to be a stress response. However, by 96 h, no significant difference in the heartbeat count was recorded between CuNPs and CuCl_2_ treatments, except for the 50 and 100 µg/L CuNPs groups. This is in line with the findings indicating decreased heart rates in the zebrafish embryos exposed to CuO NPs over 96 h [40]. Similarly, Kalishwaralal et al. [41] found a significant decrease in heart rate in zebrafish embryos exposed to higher doses of selenium-based NPs (SeNPs, 20 and 25 µg/mL).

**Figure 5:**
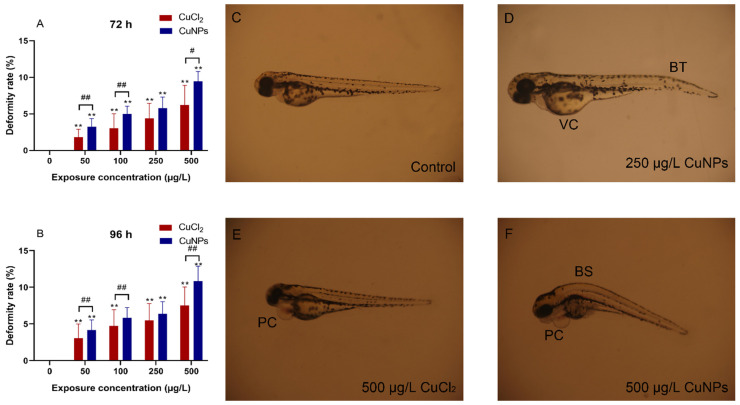
Deformity rate of zebrafish embryos exposed to CuCl_2_ and CuNPs for 96 h. (**A**) Dose-related variations in zebrafish embryo malformations at 72 h. (**B**) Dose-related variations in zebrafish embryo malformations at 96 h (except for 100 µg/L CuNPs group). (**C**–**F**) Representative optical images of deformed zebrafish. Data are mean ± SD, N = 60. ** Denotes significant difference versus the control (*p* < 0.01). # Denotes significant difference between CuCl_2_ and CuNPs groups (# *p* < 0.05, ## *p* < 0.01). The typical malformations including bent tail (BT), bent spine (BS), pericardial cyst (PC), and vitelline cyst (VC).

In general, organ dysfunction often correlates with structural damage. In this study, a pericardial cyst, indicative of heat malformations, in the zebrafish embryos was observed in both CuNPs and CuCl_2_ treatments (Figure 5). This finding is consistent with the heart rate results shown in Figure 5. In addition, various abnormalities in the developing embryos, including bent spines (BSs), bent tail (BTs), and vitelline cysts (VCs), were also recorded. The deformity rate increased with higher exposure concentration, with significant differences observed between CuNPs and CuCl_2_ treatments at concentrations of 50, 100, and 500 µg/L (*p* < 0.05). Numerous studies have reported deformities in zebrafish induced by nanomaterials [41,42,43]. For example, zebrafish embryos treated with CuO NPs exhibited sacculi or otolith, head malformation, heart malformation, yolk deformity, end tail malformation, spinal cord malformation, rachischisis, scoliosis, and tail malformation, with more intense deformities noted at higher concentration of CuO NPs [41]. Li et al. [42] found shorter spinal column, body length, and tail curvature in embryos treated with the Ag nanoplates or Ag nanospheres at the 24 hpf (hours post fertilization) stage, along with increased pericardial sac edema in both treatments at the 48 hpf stage.

### 2.3. Neurotoxicity Test

**Figure 6:**
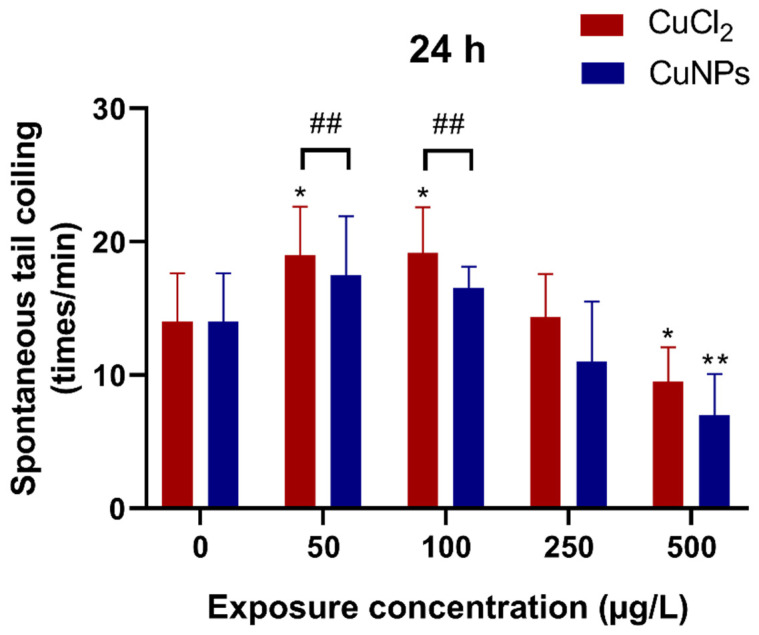
Spontaneous tail coiling of zebrafish embryos after treatment with different concentrations of CuCl_2_ and CuNPs for 24 h. Data are mean ± SD, N = 60. * Denotes significant difference versus the control (* *p* < 0.05, ** *p* < 0.01). ## Denotes significant difference between CuCl_2_ and CuNPs groups (*p* < 0.01).

Spontaneous tail coiling is the first motor behavior that could be observed in zebrafish embryos, characterized by lateral contractions of the trunk. This behavior starts around 17 hpf, peaks around 19 hpf, and subsequently decreases gradually by 26 hpf, transitioning into different motor activities [44,45]. In the present study, observation of spontaneous tail coiling in zebrafish embryos at 24 hpf revealed a slight increase in coiling frequency in the 50 and 100 µg/L CuCl_2_ treatments (*p* < 0.05) (Figure 6). Conversely, a significant reduction in coiling frequency was noted in the 500 µg/L CuNPs and CuCl_2_ treatments (*p* < 0.05), indicating potential nervous system damage from the nanomaterials. Similarly, a study on the toxicity of copper hydroxide (Cu(OH)_2_) nanopesticide observed an ~2-fold decrease in spontaneous tail coiling in embryos exposed to both Cu(OH)_2_ nanopesticide and Cu^2+^ at 100 μg/L. Their subsequent study revealed alterations in neurotransmitter-related pathways (dopaminergic, serotoninergic, GABAergic, glutamatergic) following Cu(OH)_2_ nanopesticide exposure. These changes in neurotransmitter-related pathways are suggested to underlie the observed behavioral shifts in zebrafish [46].

**Figure 7:**
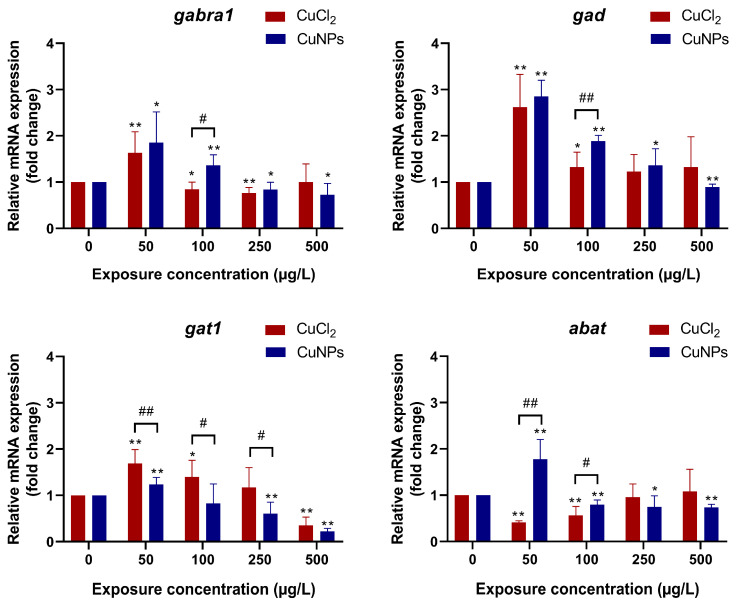
mRNA expression of GABA signaling pathway-related genes in zebrafish embryos after treatment with different concentrations of CuCl_2_ and CuNPs for 96 h. The experiment was performed in sextuplicate with N = 30 for each treatment. Data are mean ± SD. * D significant difference versus the control (* *p* < 0.05, ** *p* < 0.01). # Denotes significant difference between CuCl_2_ and CuNPs groups (# *p* < 0.05, ## *p* < 0.01).

γ-Aminobutyric acid (GABA) is one of most important inhibitory neurotransmitters within the central nervous system [47]. Upon release from the presynaptic membrane, GABA engages GABAA and/or GABAB receptors, consequently inhibiting neurotransmitter release and neuronal activity [48,49]. In the present study, the transcriptional expression of key genes involved in the GABA signaling pathway were investigated in zebrafish embryos exposed to CuNPs and CuCl_2_. As shown in Figure 7, the transcriptional expression of GABAA receptor alpha 1 (*gabra1*) was upregulated by low concentrations of CuNPs (50 and 100 µg/L) and CuCl_2_ (50 µg/L), while it was notably downregulated (*p* < 0.05) by high doses of CuNPs (250 and 500 µg/L) and CuCl_2_ (100 and 250 µg/L). It is well known that GABA typically exerts its inhibitory effects by binding to postsynaptic GABA receptors [48], and the alteration in *gabra1* mRNA expression may indicate a disruption in GABA-receptor receptors. Furthermore, CuNPs or CuCl_2_ exposure also regulated the transcriptional expression of genes related to GABA biosynthesis, catabolism, and transport (Figure 7). GABA is synthesized through glutamic acid decarboxylation catalyzed by glutamate decarboxylase (GAD). After CuNPs or CuCl_2_ exposure, the mRNA expression of *gad* varied, generally increasing except for a decrease observed with 500 µg/L CuNPs. It was speculated that the enhancement in *gad* mRNA expression might lead to the increased GABA accumulation. In addition, GABA can be secreted from neurons or glial cells through GABA transporter (GAT) reversal [50]. Consequently, the increase in *gat1* mRNA expression following a low-concentration CuNPs or CuCl_2_ exposure might elevate the GABA level. However, *gat1* mRNA expression significantly decreased at 500 µg/L CuNPs and CuCl_2_ (Figure 7). GABA transporters, such as GAT-1 in neurons and GAT-3 in glia, regulate synaptic GABA release, thereby modulating its inhibitory effects. GABA can be recycled by vesicular uptake into a presynaptic membrane or reuptake into a gliocyte. Then, it is catabolized to succinic semialdehyde and glutamate via GABA-transaminase (GABA-T) [51]. In the present study, the mRNA expression of *abat*, encoding GABA-T, was significantly reduced (*p* < 0.05) by CuNPs exposure, except at 50 µg/L CuNPs (Figure 7). It was implied that the catabolism of GABA was inhibited. Consequently, disrupted GABA release, receptor interaction, and neurotransmitter conduction could contribute to altered neurobehavior, such as the observed decline in spontaneous tail coiling frequency (Figure 6). In the exposure experiment involving Cu(OH)_2_ nanopesticide, glutamate decarboxylase 1b (*gad1b*) mRNA expression in the zebrafish was reduced, alongside differential expression of GABA receptors (*gabra2a*, *gabra5*, *gabrr1*) and other genes (*grin2b*, *gabra2a*, *gabrb2a*) following nanopesticide and copper ion exposures [46].

### 2.4. Oxidative Stress and Inflammatory Response

**Figure 8:**
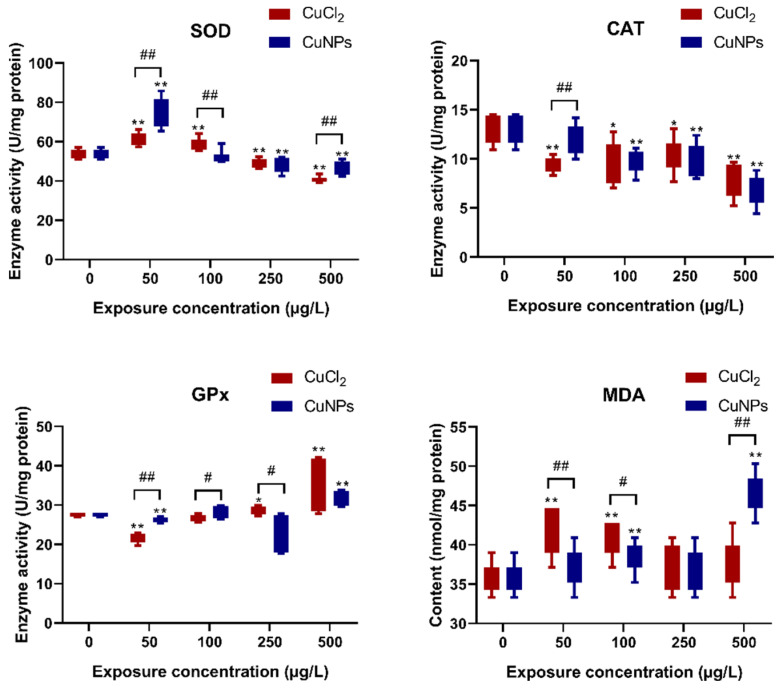
Activity of antioxidative enzymes in zebrafish embryos after treatment with different concentrations of CuCl_2_ and CuNPs for 96 h. The experiment was performed in triplicate with N = 60 for each treatment. Data are mean ± SD. * Denotes significant difference versus the control (* *p* < 0.05, ** *p* < 0.01). # Denotes significant difference between CuCl_2_ and CuNPs groups (# *p* < 0.05, ## *p* < 0.01).

Most studies have indicated that the generation of reactive oxygen species (ROS) and subsequent oxidative stress are commonly observed in the NP-induced toxicity [52]. NP-mediated ROS responses are thought to be linked to a range of pathological outcomes such as inflammation, genotoxicity, carcinogenesis, and fibrosis [53]. In the present study, the activity of antioxidative enzymes and the levels of malondialdehyde (MDA) were tested to evaluate the oxidative stress induced by CuNPs or CuCl_2_. Our results showed that superoxide dismutase (SOD) activity was induced at doses of 50 and 100 µg/L of CuNPs or CuCl_2_ but inhibited at doses of 250 and 500 µg/L of CuNPs and CuCl_2_ (Figure 8). Additionally, the activities of glutathione peroxidase (GPx) and catalase (CAT) were generally decreased, except at 500 µg/L CuCl_2_. Moreover, the MDA content increased significantly in most treatments (*p* < 0.01). It was inferred that exposure to high concentrations of CuNPs or CuCl_2_ could lead to reduced antioxidative enzyme activity, resulting in ROS accumulation and subsequent lipid peroxidation. The decline in antioxidative enzyme activity at high concentrations may be due to impaired synthesis or structure changes in enzyme proteins induced by exposure to CuNPs or CuCl_2_, which resulted in the enzymes’ inactivation. Similarly, oxidative stress in the liver, gills, and head of the zebrafish induced by zinc oxide nanoparticles (Nano-ZnO) and perfluorooctanesulfonic acid potassium salt (PFOS) were investigated [54]. The results showed that both Nano-ZnO and PFOS, whether in co-exposure or single exposure, triggered oxidative stress. In co-exposure solutions, the CAT activity was significantly lower, but the MDA content was remarkably higher compared to single exposure.

**Figure 9:**
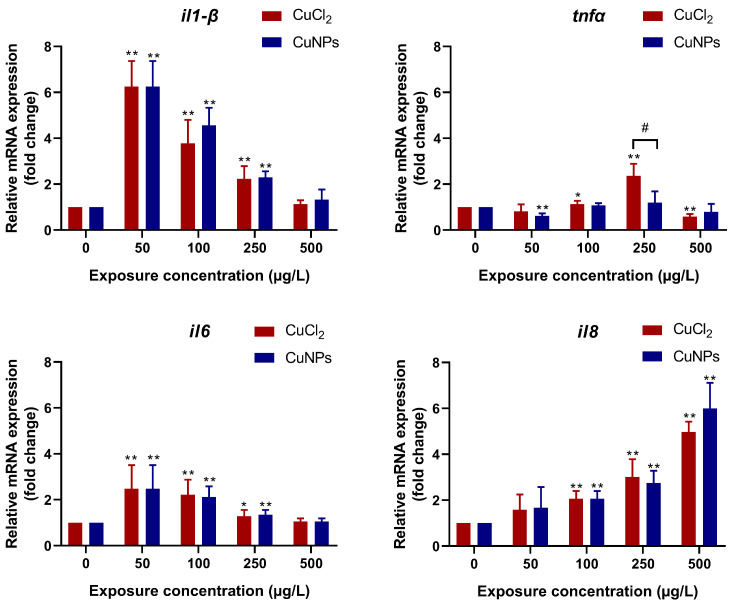
mRNA expression of inflammatory factors in zebrafish embryos after treatment with different concentrations of CuCl_2_ and CuNPs for 96 h. The experiment was performed in sextuplicate with N = 30 for each treatment. Data are mean ± SD. * Denotes significant difference versus the control (* *p* < 0.05, ** *p* < 0.01). # Denotes significant difference between CuCl_2_ and CuNPs groups (*p* < 0.05).

Studies have revealed the interdependent relationship between ROS and inflammation upon exposure to NPs. Inflammatory cells like neutrophils and macrophages cause abundant ROS release to eliminate the NPs [55]. However, NP-exposure-induced oxidative stress activates MAPK, RTK, NF-*κ*B, and Akt, leading to a proinflammatory cascade [56]. The results indicated that the mRNA expression of inflammatory factors was regulated by CuNPs and CuCl_2_ exposure (Figure 9). Proinflammatory cytokines like IL-1β, IL-6, and TNF-α are commonly assessed to estimate the immunotoxicity of nanomaterials [57]. Changes in the mRNA expression of these cytokines in the present study provided evidence of immunotoxicity associated with CuNPs and CuCl_2_ exposure. It is speculated that CuNPs- or CuCl_2_-induced oxidative stress triggered the proinflammatory cascade, as evidenced by the enhanced transcriptional expression of *il-1β*, *tnf-α*, *il-6,* and *il-8* to varying degrees. Similar findings have been reported in in vitro studies. Transcriptome data showed the inflammatory gene expression in astrocytes repeatedly exposed to micro- and nanoplastics (MNPLs) for 168 h. The transcriptional expression of *il-1β*, *tnf,* and *il-6* was upregulated by MNPLs of different sizes (50 nm and 500 nm) [58]. Wang et al. [59] explored the proinflammatory effect and cytotoxicity of PS nanoplastics (PS-NPs) and PS microplastics (PS-MPs) on mouse macrophage RAW264.7 cells. They observed that the release of inflammatory cytokines (TNF-α, Il-6, and Il-10) was increased by 1, 0.01, and 0.01 μg/mL PS-NPs, and the contents of TNF-α, Il-6, and Il-10 were also enhanced by 1 and 0.01 μg/mL PS-MPs.

## 3. Materials and Methods

### 3.1. Zebrafish Breeding

The parental zebrafish (AB wild-type strain) were obtained from the China Zebrafish Resource Center (CZRC) and cultured in a circulating filtration system under controlled conditions (28 ± 1 °C, 14 h light/10 h dark photoperiod). To acclimate the fish to laboratory conditions, males and females were fed with brine shrimp twice daily for one month. In the evening, two males and two females were randomly selected and transferred into breeding tanks. The next morning, the embryos were collected, washed three times with RO (reverse osmosis) water, and then placed in 100 mm glass Petri dish with 60 embryos per dish for exposure. This research received approval from the Committee of Scientific Research in Shanxi University (CSRSX, approval No. SXULL2024009), and all the zebrafish protocols were performed according to guidelines from the CSRSX.

### 3.2. Exposure Protocols

Copper chloride (CuCl_2_, analytically pure) was purchased from Tianjin Bondi Chemical Co., Ltd. (Tianjin, China). The CuNPs colloid was purchased from XFNano Technology Co., Ltd. (Cat.No.: 100420, Nanjing, China), dispersed in ultrapure water through serial dilutions, and sonicated (SB-5200DTDN, Scientz Biotechnology Co., Ningbo, China) for 30 min prior to introduction to the exposure groups. Transmission electron microscopy (TEM) images were taken with a JEM-1011 Transmission Electron Microscope (JEOL, Tokyo, Japan), revealing that CuNPs used in this research were spherical particles of approximately 5–10 nm, consistent with the manufacturer’s description.

The exposure concentrations were chosen according to the aquatic environmental concentration of copper [60,61] and the reported lethal dose of 50% (LC_50_) of CuNPs in aquatic animals [22]. Stock solutions of CuNPs and copper ions were individually prepared and diluted by RO water to achieve copper concentrations of 50, 100, 250, and 500 μg/L. RO water served as the control. The exposure duration was 96 h, and fresh exposure solutions were prepared and replaced daily. Dead embryos were removed from the exposure solution each day. The mortality rates were recorded every 24 h. Spontaneous tail coiling of zebrafish embryos at 24 hpf was analyzed. The hatching rate and heart rate were recorded at 48, 72, and 96 hpf. The deformity rate was observed with a stereomicroscope (SZX16, OLYMPUS, Tokyo, Japan) and calculated at 72 and 96 hpf.

### 3.3. Measurement of Copper Bioaccumulation

Thirty larvae exposed for 96 h were collected from each treatment and rinsed with deionized water to remove the CuCl_2_/CuNPs adhering to the surface. Subsequently, the samples were dried at 80 °C until reaching a constant weight. The Cu concentration within the larvae was measured using inductively coupled plasma mass spectrometry (ICP-MS, NexION 350, PerkinElmer, Hopkinton, MA, USA) following acid digestion with HNO_3_, employing the microwave digestion technique.

### 3.4. Physiological Parameter Analysis

The zebrafish embryos were observed at intervals of 24 h until 96 hpf. The mortality rate, hatching rate, heart rate, deformity rate, and frequency of spontaneous tail coiling were recorded at each 24 h interval [62]. The hatching rate and heart rate of each individual were observed at 48, 72, and 96 hpf. The heart rate was recorded by counting the number of beats that occurred over a 20 sec interval. Then, it was extrapolated to 1 min to indicate the average beats per minute [63]. Morphological abnormalities such as the presence of bent tail, bent spine, pericardial edema, and yolk sac edema were observed by a stereomicroscope at 4× magnification (SZX16, OLYMPUS, Tokyo, Japan). The frequency of spontaneous tail coiling (times/min) was investigated in zebrafish embryos at 24 hpf. 

### 3.5. Enzymes Activity Analysis

Sixty larvae were collected from each treatment and homogenized with an ultrasonic cell crusher (JY98-IIIN, SCIENTZ, Ningbo, China) under ice-cold conditions, with the addition of Tris-HCl buffer (*w*/*v*, 1:9, pH 7.4, 0.01 mol/L, containing 0.01 mol/L of sucrose, 0.0001 mol/L of EDTA-2Na, and 0.8% of NaCl) [21]. The homogenates were then centrifuged, and the resulting supernatant was collected to measure the oxidative stress parameters. The activities of SOD, CAT, and GPx, as well as the content of MDA, were measured using the commercial reagent kits from Nanjing Jiancheng Bioengineering Institute (Nanjing, China), according to the manufacturer’s instructions. The absorbance reading was taken using a spectrophotometer (SpectraMax M5, Molecular Devices, San Jose, CA, USA).

### 3.6. mRNA Expression Analysis

Zebrafish at 96 hpf from the control group, CuNPs treatment, and CuCl_2_ treatment were collected. Thirty larvae were pooled in a centrifugal tube as one sample, and six replicates were prepared for each group. The total RNA of each sample was isolated with RNAiso Plus from Takara (Tokyo, Japan), according to the manufacturer’s instructions and quantified using a microspectrophotometer (Eppendorf, Hamburg, Germany). One microgram of purified total RNA from each sample was reverse-transcribed into cDNA by PrimeScript RT Master Mix (Takara, Tokyo, Japan).

The PCR amplification was performed on 7500 Real-Time PCR Systems (Applied Biosystems, Wakefield, RI, USA) with TB Green Premix Ex TaqII (Takara, Tokyo, Japan). Six replicates were run in triplicate for each treatment. The results of real-time fluorescence quantitative PCR were normalized using *rpl7* as the housekeeping gene, and the Ct value was converted into relative expression (fold change) using the 2^−ΔΔCt^ method. The primer sets are listed in Table 1.

### 3.7. Statistical Analysis

Graph-Pad Prism (version 8.0) was used for statistics and data visualization. The data were shown as mean ± SD (standard deviation). One-way analysis of variance (ANOVA) followed by the Tukey test was applied for multiple comparisons. A significance level of *p* < 0.05 was considered statistically significant.

## 4. Conclusions

In summary, the findings of this study suggest that exposure to CuNPs can lead to an increase in the death and deformity rates, as well as a decline in heart rate and the frequency of spontaneous tail coiling in zebrafish embryos. These results indicate the toxic effects of CuNPs on the development of zebrafish embryos. In addition, the changes in the transcriptional expression of key genes in the GABA signaling pathway suggest that CuNPs may interfere with the synthesis, release, catabolism, and recovery of GABA, potentially leading to disruptions in neurotransmitter conduction and neurobehavioral disorder. Furthermore, the inhibition of antioxidative enzyme activities and the increase in MDA content demonstrate the oxidative stress caused by CuNPs exposure. This oxidative stress, in turn, triggers a proinflammatory cascade. Comparatively, CuCl_2_ exhibited more severe toxic effects on zebrafish embryos across most of the tested parameters in this study than CuNPs did. However, further research is required to explore the long-term toxic effects of CuNPs and the associated mechanisms of toxicity.

## Figures and Tables

**Table 1:** Primers used for real-time PCR analysis.

Gene		Primer (5′-3′)	Genbank No.	Product Length (bp)
*rpl7*	F	CAGAGGTATCAATGGTGTCAGCCC	NM_213213644.2	119
	R	TTCGGAGCATGTTGATGGAGGC		
*il-1β*	F	TGATGAATGGGTGTTCGGCA	NM_012799.1	248
	R	TACCGATGCGTGCTACTTCC		
*tnf-α*	F	CTCCATAAGACCCAGGGCAA	NM_212859.2	162
	R	CTGGTCCTGGTCATCTCTCC		
*il-6*	F	GCAAGAACGAAAACTGCAGC	NM_001079833.1	182
	R	CGATTCAGTCTGACCGGAGA		
*il-8*	F	TGTGTTATTGTTTTCCTGGCATTTC	XM_009306855.1	81
	R	GCGACAGCGTGGATCTACAG		
*gabra1*	F	TCAGGCAGAGCTGGAAGGAT	NM_001077326	117
	R	TGCCGTTGTGGAAGAACGT		
*abat*	F	GCGTTCAGGCAAAGCTCT	NM_201498	204
	R	GCAGGACGGAAACGGAT		
*gad*	F	AACTCAGGCGATTGTTGCAT	NM_194419	109
	R	TGAGGACATTTCCAGCCTTC		
*gat1*	F	GTGCGAGGACAAGTGCAAAG	NM_001007362	193
	R	ATGTTTGGAGGTTTGGGGCA		

## Data Availability

Data are contained within the article.

## References

[1] Yang J., Hu S., Rao M., Hu L., Lei H., Wu Y., Wang Y., Ke D., Xia W., Zhu C. (2017). Copper nanoparticle-induced ovarian injury, follicular atresia, apoptosis, and gene expression alterations in female rats. Int. J. Nanomed..

[2] Deka P., Borah B.J., Saikia H., Bharali P. (2019). Cu-based nanoparticles as emerging environmental catalysts. Chem. Rec..

[3] Zhang C., Wang Y., Sun Q., Xia L., Hu J., Cheng K., Wang X., Fu X., Gu H. (2018). Copper nanoparticles show obvious in vitro and in vivo reproductive toxicity via ERK mediated signaling pathway in female mice. Int. J. Biol. Sci..

[4] Ameh T., Sayes C.M. (2019). The potential exposure and hazards of copper nanoparticles: A review. Environ. Toxicol. Pharmacol..

[5] Sirotkin A.V., Radosová M., Tarko A., Martín-García I., Alonso F. (2020). Effect of morphology and support of copper nanoparticles on basic ovarian granulosa cell functions. Nanotoxicology.

[6] Crisan M.C., Teodora M., Lucian M. (2022). Copper nanoparticles: Synthesis and characterization, physiology, toxicity and antimicrobial applications. Appl. Sci..

[7] Chung K., Bang J., Thacharon A., Song H.Y., Kang S.H., Jang W., Dhull N., Thapa D., Ajmal C.M., Song B. (2022). Non-oxidized bare copper nanoparticles with surface excess electrons in air. Nat. Nanotechnol..

[8] Zou L., Cheng G., Xu C., Liu H., Wang Y., Li N., Fan X., Zhu C., Xia W. (2021). Copper nanoparticles induce oxidative stress via the heme oxygenase 1 signaling pathway in vitro studies. Int. J. Nanomed..

[9] Ahamed M., Lateef R., Akhtar M.J., Rajanahalli P. (2022). Dietary antioxidant curcumin mitigates CuO nanoparticle-induced cytotoxicity through the oxidative stress pathway in human placental cells. Molecules.

[10] Luisa M., Giacomo C. (2014). Acute toxicity test of CuO nanoparticles using human mesenchymal stem cells. Toxicol. Mech. Methods.

[11] Mahmood R.I., Kadhim A.A., Ibraheem S., Albukhaty S., Mohammed-Salih H.S., Abbas R.H., Jabir M.S., Mohammed M.K., Nayef M., AlMalki F.A. (2022). Biosynthesis of copper oxide nanoparticles mediated *Annona muricata* as cytotoxic and apoptosis inducer factor in breast cancer cell lines. Sci. Rep..

[12] Anima B., Mondal P., Gurusubramanian G., Roy V.K. (2023). Mechanistic study of copper nanoparticle (CuNP) toxicity on the mouse uterus via apelin signaling. Environ. Sci. Pollut. Res. Int..

[13] Pohanka M. (2019). Copper and copper nanoparticles toxicity and their impact on basic functions in the body. Bratisl. Med. J..

[14] Abbasi A., Ghorban K., Nojoomi F., Dadmanesh M. (2021). Smaller copper oxide nanoparticles have more biological effects versus breast cancer and nosocomial infections bacteria. Asian Pac. J. Cancer Prev..

[15] Jiang W., Kim B.Y.S., Rutka J.T., Chan W.C.W. (2008). Nanoparticle-mediated cellular response is size-dependent. Nat. Nanotech..

[16] Høgset H., Horgan C.C., Armstrong J.P.K., Bergholt M.S., Torraca V., Chen Q., Keane T.J., Bugeon L., Dallman M.J., Mostowy S. (2020). In vivo biomolecular imaging of zebrafish embryos using confocal Raman spectroscopy. Nat. Commun..

[17] Haque E., Ward A.C. (2018). Zebrafish as a model to evaluate nanoparticle toxicity. Nanomaterials.

[18] Strähle U., Scholz S., Geisler R., Greiner P., Hollert H., Rastegar S., Schumacher A., Selderslaghs I., Weiss C., Witters H. (2012). Zebrafish embryos as an alternative to animal experiments—A commentary on the definition of the onset of protected life stages in animal welfare regulations. Reprod. Toxicol..

[19] Gagné F., André C., Skirrow R., Gélinas M., Auclair J., Aggelen G., Turcott P., Gagnon C. (2012). Toxicity of silver nanoparticles to rainbow trout: A toxicogenomic approach. Chemosphere.

[20] Cambier S., Rogeberg M., Georgantzopoulou A., Serchi T., Karlsson C., Verhaegen S., Iversen T.G., Guignard C., Kruszewski M., Hoffmann L. (2018). Fate and effects of silver nanoparticles on early life-stage development of zebrafish (*Danio rerio*) in comparison to silver nitrate. Sci. Total Environ..

[21] Yang L., He Z., Li X., Jiang Z., Xuan F., Tang B., Bian X. (2022). Behavior and toxicity assessment of copper nanoparticles in aquatic environment: A case study on red swamp crayfish. J. Environ. Manag..

[22] Yang L., Wang W. (2019). Comparative contributions of copper nanoparticles and ions to copper bioaccumulation and toxicity in barnacle larvae. Environ. Pollut..

[23] Wang T., Long X.H., Cheng Y., Liu Z., Yan S. (2014). The potential toxicity of copper nanoparticles and copper sulphate on juvenile *Epinephelus coioides*. Aquat. Toxicol..

[24] Yang Y., Qin Z., Zeng W., Yang T., Cao Y., Mei C., Kuang Y. (2017). Toxicity assessment of nanoparticles in various systems and organs. Nanotechnol. Rev..

[25] Bostan H.B., Rezaee R., Valokala M.G., Tsarouhas K., Golokhvast K., Tsatsakis A.M., Karimi G. (2016). Cardiotoxicity of nano-particles. Life Sci..

[26] Brun N., Varela M., Peijnenburg W.J.G.M., Spaink H.P., Koch B.E.V., Vijver M.G. (2018). Nanoparticles induce dermal and intestinal innate immune system responses in zebrafish embryos. Environ. Sci. Nano.

[27] Ates M., Demir V., Adiguzel R., Arslan Z. (2013). Bioaccumulation, subacute toxicity, and tissue distribution of engineered titanium dioxide nanoparticles in goldfish (*Carassius auratus*). J. Nanomater..

[28] Stainier D.Y., Fishman M.C. (1994). The zebrafish as a model system to study cardiovascular development. Trends Cardiovasc. Med..

[29] Dooley K., Zon L.I. (2000). Zebrafish: A model system for the study of human disease. Curr. Opin. Genet. Dev..

[30] Jurewicz A., Ilyas S., Uppal J.K., Ivandic I., Korsching S., Mathur S. (2020). Evaluation of magnetite nanoparticle-based toxicity on embryo–larvae stages of zebrafish (*Danio rerio*). ACS Appl. Nano Mater..

[31] Lee W.S., Cho H.J., Kim E., Huh Y.H., Kim H., Kim B., Kang T., Lee J.S., Jeong J. (2019). Bioaccumulation of polystyrene nanoplastics and their effect on the toxicity of Au ions in zebrafish embryos. Nanoscale.

[32] Powers C.M., Yen J., Linney E.A., Seidler F.J., Slotkin T.A. (2010). Silver exposure in developing zebrafish (*Danio rerio*): Persistent effects on larval behavior and survival. Neurotoxicol. Teratol..

[33] Clemente Z., Castro V.L., Moura M.A., Jonsson C.M., Fraceto L.F. (2014). Toxicity assessment of TiO_2_ nanoparticles in zebrafish embryos under different exposure conditions. Aquat. Toxicol..

[34] Ahmad F., Liu X., Zhou Y., Yao H. (2015). An in vivo evaluation of acute toxicity of cobalt ferrite (CoFe_2_O_4_) nanoparticles in larval-embryo Zebrafish (*Danio rerio*). Aquat. Toxicol..

[35] Wang Y., Zhou J., Liu L., Huang C., Zhou D., Fu L. (2016). Characterization and toxicology evaluation of chitosan nanoparticles on the embryonic development of zebrafish, *Danio rerio*. Carbohydr. Polym..

[36] Samaee S.M., Rabbani S., Jovanović B., Mohajeri-Tehrani M.R., Haghpanah V. (2015). Efficacy of the hatching event in assessing the embryo toxicity of the nano-sized TiO_2_ particles in zebrafish: A comparison between two different classes of hatching-derived variables. Ecotoxicol. Environ. Saf..

[37] Caro C., Egea-Benavente D., Polvillo R., Royo J.L., Leal P.M., García-Martín M.L. (2019). Comprehensive toxicity assessment of PEGylated magnetic nanoparticles for in vivo applications. Colloids. Surf. B Biointerfaces.

[38] Priyam A., Singh P.P., Afonso L.B., Schultz A.G. (2022). Exposure to biogenic phosphorus nano-agromaterials promotes early hatching and causes no acute toxicity in zebrafish embryos. Environ. Sci. Nano.

[39] De Luca E., Zaccaria G., Hadhoud M., Rizzo G., Ponzini R., Morbiducci U., Santoro M.M. (2014). ZebraBeat: A flexible platform for the analysis of the cardiac rate in zebrafish embryos. Sci. Rep..

[40] Ganesan S., Thirumurthi N.A., Raghunath A., Vijayakumar S., Perumal E. (2016). Acute and sub-lethal exposure to copper oxide nanoparticles causes oxidative stress and teratogenicity in zebrafish embryos. J. Appl. Toxicol..

[41] Kalishwaralal K., Jeyabharathi S., Sundar K., Muthukumaran A. (2016). A novel one-pot green synthesis of selenium nanoparticles and evaluation of its toxicity in zebrafish embryos. Artif. Cells Nanomed. Biotechnol..

[42] Li K., Zhao X., Zhai Y., Chen G., Lee E.H., He S. (2015). A study on the biocompatibility of surface-modified Au/Ag alloyed nanobox particles in zebrafish in terms of mortality rate, hatch rate and imaging of particle distribution behavior. Prog. Electromagn. Res..

[43] Duan J., Yu Y., Shi H., Tian L., Guo C., Huang P., Zhou X., Peng S., Sun Z. (2013). Toxic effects of silica nanoparticles on zebrafish embryos and larvae. PLoS ONE.

[44] Oliveira A.A.S., Brigante T.A.V., Oliveira D.P. (2021). Tail coiling assay in zebrafish (*Danio rerio*) embryos: Stage of development, promising positive control candidates, and selection of an appropriate organic solvent for screening of developmental neurotoxicity (DNT). Water.

[45] Saint-Amant L., Drapeau P. (1998). Time course of the development of motor behaviors in the zebrafish embryo. J. Neurobiol..

[46] Chen S., Qin Y., Ye X., Liu J., Yan X., Zhou L., Wang X., Martyniuk C.J., Yan B. (2023). Neurotoxicity of the Cu(OH)_2_ nanopesticide through perturbing multiple neurotransmitter pathways in developing zebrafish. Environ. Sci. Technol..

[47] Jayakumar A.R., Sujatha R., Paul V., Asokan C., Govindasamy S., Jayakumar R. (1999). Role of nitric oxide on GABA, glutamic acid, activities of GABA-T and GAD in rat brain cerebral cortex. Brain Res..

[48] Ghit A., Assal D., Al-Shami A.S., Hussein D.E. (2021). GABAA receptors: Structure, function, pharmacology, and related disorders. J. Genet. Eng. Biotechnol..

[49] Huang Z.J. (2009). Activity-dependent development of inhibitory synapses and innervation pattern: Role of GABA signaling and beyond. J. Physiol..

[50] Koch U., Magnusson A.K. (2009). Unconventional GABA release: Mechanisms and function. Curr. Opin. Neurobiol..

[51] Jin X., Galvan A., Wichmann T., Smith Y. (2011). Localization and function of GABA transporters GAT-1 and GAT-3 in the basal ganglia. Front. Syst. Neurosci..

[52] Li N., Xia T., Nel A.E. (2008). The role of oxidative stress in ambient particulate matter-induced lung diseases and its implications in the toxicity of engineered nanoparticles. Free Radical Bio. Med..

[53] Li J.J., Muralikrishnan S., Ng C.T., Yung L.Y., Bay B.H. (2010). Nanoparticle-induced pulmonary toxicity. Exp. Biol. Med..

[54] Liu Z., Du J., Wang S., You H. (2016). PFOS and ZnO nanoparticles induced oxidative stress in liver, gill and head of zebrafish. Sci. Discov..

[55] Manke A., Wang L., Rojanasakul Y. (2013). Mechanisms of nanoparticle-induced oxidative stress and toxicity. BioMed Res. Int..

[56] Donaldson K., Poland C.A. (2012). Inhaled nanoparticles and lung cancer—What we can learn from conventional particle toxicology. Swiss Med. Wkly..

[57] Lina T.T., Johnson S.J., Wagner R.D. (2020). Intravaginal poly-(D, L-lactic-co-glycolic acid)-(polyethylene glycol) drug-delivery nanoparticles induce pro-inflammatory responses with Candida albicans infection in a mouse model. PLoS ONE.

[58] Marcellus K.A., Bugiel S., Nunnikhoven A., Curran I., Gill S.S. (2024). Polystyrene nano- and microplastic particles induce an inflammatory gene expression profile in rat neural stem cell-derived astrocytes in vitro. Nanomaterials.

[59] Wang X., Ren X., He H., Li F., Liu K., Zhao F., Hu H., Zhang P., Huang B., Pan X. (2023). Cytotoxicity and pro-inflammatory effect of polystyrene nano-plastic and micro-plastic on RAW264.7 cells. Toxicology.

[60] Zuo H., Ma X., Yang K., Chen Y., Chen J., Guo Y., Zhao J., Wang R., Fang F., Liu Y. (2016). distribution and risk assessment of metals in surface water and sediment in the upper reaches of the Yellow River, China. Soil. Sediment. Contam..

[61] Čmelík J., Brovdyová T., Trögl J., Neruda M., Kadlečík M., Pacina J., Popelka J., Sirotkin A. (2019). Changes in the content of heavy metals in Bílina River during 2012–2017: Effects of flood and industrial inputs. Water.

[62] Abdullah S.N.S., Subramaniam K.A., Zamani Z.H.M., Sarchio S.N.E., Yasin F.M., Shamsi S. (2022). Biocompatibility study of curcumin-loaded pluronic F127 nanoformulation (NanoCUR) against the embryonic development of zebrafish (*Danio rerio*). Molecules.

[63] Shamsi S., Alagan A.A., Sarchio S.N.E., Yasin F.M. (2020). Synthesis, characterization, and toxicity assessment of Pluronic F127-functionalized graphene oxide on the embryonic development of zebrafish (*Danio rerio*). Int. J. Nanomed..

